# Transition to electronic prescriptions in pharmacies: Workflows, services, and access to medication – A mixed methods approach

**DOI:** 10.1016/j.rcsop.2026.100706

**Published:** 2026-01-25

**Authors:** Alexander Graf, Maike Henningsen, Maximillian Zinner

**Affiliations:** Witten/Herdecke University, School of Medicine, Faculty of Health, Alfred Herrhausen-Strasse 50, 58448 Witten, Germany

**Keywords:** Electronic prescribing, Medication dispensing, (Digital) pharmacy services, Access to medication, Technology acceptance

## Abstract

**Background:**

Germany's declining number of pharmacies raised concerns about patients' access to medication. To address this, pharmacies have started offering (digital) services, including analog and digital options such as phone consultation or medication delivery. According to existing literature, these services could improve access to medication. However, before the introduction of electronic prescriptions (ePs) in 2022, using these services in Germany was cumbersome.

**Objectives:**

Given this context, the aim of the study was to examine the challenges and potentials of electronic prescriptions in German pharmacies.

**Methods:**

A two-stage sequential mixed methods approach was used, combining semi-structured interviews with an online survey among 1215 pharmacists.

**Results:**

Nearly half of surveyed pharmacists (49.3%) supported the transition to electronic prescriptions, while 37.0% opposed it. Technical errors were widespread (mentioned by 90.5%) and disrupted both dispensing and service workflows. Over half (53.3%) believed ePs worsened in-pharmacy pick-up for patients, while 59.3% saw no major effect on (digital) services. More than one in four (28.5%) observed improvements in medication delivery through digital transfer, but many noted increased competition from large online pharmacies, increased workflow complexity, limited perceived patient demand and low profitability.

**Conclusion:**

Electronic prescriptions were associated with many challenges for pharmacists. Although they might have the potential to improve patient access to medications, this remains limited by technical instability, low patient digital literacy, and pharmacists' perceptions of limited usefulness. Enhancing pharmacist and patient experiences by reducing technical errors, ensuring profitability of (digital) services, and improving patients' readiness is essential to realize the potential of ePs.

## 1 Introduction

Germany's declining number of pharmacies has raised concerns about patients' access to medication.^1^ Between 2013 and 2022, the number of pharmacies fell by 12.6% to 18,000, which is lower than in 1980, resulting in a pharmacy density 31% below the European Union (EU) average number of pharmacies per inhabitant.^2^ Key drivers include competitive pressure, succession challenges, and a shrinking number of pharmacy students.^3^

Although most patients still reported satisfactory access to pharmacies, experts anticipate a strong decline in accessibility,^3^ particularly in less populated areas, where access to pharmacies is already limited.^4^ At the same time, the number of annual prescriptions has increased in recent years,^5^ and medication shortages^6^ have forced patients to make multiple trips to pharmacies. To counter these challenges, pharmacies started offering (digital) services such as medication delivery, preordering, and click & collect.^7^ These services may improve access to medication, especially through postal medication delivery.^8^, ^9^, ^10^

Postal medication delivery has been allowed in Germany since 2004, and by 2021, approximately 15% of pharmacies held a delivery license.^11^ Before the introduction of electronic prescriptions (ePs) in 2022,^12^ which are medical prescriptions that are issued, stored, and redeemed electronically,^13^ paper-based prescriptions made postal medication delivery cumbersome^14^: Patients had to physically mail their prescriptions to the pharmacy, which could dispense medication only after receiving the original document, often causing multi-day delays. Previous literature concluded that ePs could simplify postal medication delivery in Germany.^15^^,^^16^

The German eP system uses a central database that can be securely accessed by physicians and pharmacists. Patients receive prescriptions either fully digitally via their health insurance card or as a printed QR code, and can transfer ePs digitally to pharmacies via different apps.^17^ Appendix A.1 illustrates the different processes of traditional and eP-based postal medication delivery in Germany.

Compared to countries such as Estonia or the UK, which have long-established e-prescription systems,^10^ Germany has been slow to implement ePs. For example, in Denmark, over 88% of prescriptions are exchanged electronically.^13^

This study therefore examines the potential of ePs to improve workflow efficiencies for pharmacists, patients' service usability, and patients' access to medication in Germany.

## 2 Theory and methods

A literature review identified no suitable framework adequately addressing the research question; therefore, a mixed methods approach consisting of semi-structured qualitative interviews and a quantitative online survey with topic experts (licensed pharmacists) was used (Appendix A.2). This method followed a thematic analysis approach for developing a conceptual model.^18^

### 2.1 Qualitative interviews

Explorative, qualitative interviews were conducted to investigate pharmacy workflows, the introduction of ePs, and (digital) service offerings in Germany. Since few patients were using ePs at the time, licensed pharmacists, who are professionally trained with a university degree in pharmacy and authorized to dispense prescription medications,^19^ were identified as the most knowledgeable group.

A semi-structured interview guide (Appendix A.3) was developed based on literature about the benefits of digital technologies in healthcare,^20^^,^^21^ technology acceptance,^22^^,^^23^ and studies on ePs, both internationally^24^^,^^25^ and in Germany.^12^^,^^26^ Purposive sampling^27^ was employed to approach a diverse group of pharmacists by phone and via email. The interviews were conducted one-on-one by phone, lasting 20–45 min, and documented through extensive note-taking.

Analysis followed Braun and Clarke's thematic framework.^28^ Notes were reviewed to identify meaningful information, aggregated and categorized (e.g., benefits of ePs), and compared across interviews. After each interview round, consolidated notes were discussed among the researchers to identify emerging themes and areas to further investigate in subsequent interviews. Themes were identified through repeated discussion among the research team and reflected pharmacists' perspectives on the overall digitalization and introduction of ePs in Germany, the benefits and drawbacks of ePs for patients and pharmacists, and the effects of ePs on (digital) services in pharmacies.

Interviews were conducted until the research team concluded thematic saturation, when no substantially new insights were generated, and additional interviews only confirmed existing findings. Identified themes informed the design of the subsequent online questionnaire.

### 2.2 Quantitative online survey

The survey was developed using LimeSurvey^29^ and was pretested with nine pharmacists and researchers. Based on their feedback, adjustments were made to wording and survey flow. Content validity was ensured through expert review by these pharmacists and health services researchers, who confirmed the appropriateness and completeness of the questions and answer options.^30^ The final questionnaire (Appendix A.4) included 12 main and 9 demographic questions, organized into five thematic blocks: Digitalization of pharmacies in Germany, benefits and drawbacks of ePs for patients, benefits and drawbacks of ePs for pharmacists, (digital) service offerings utilizing ePs, and demographic information.

A five-point Likert scale was used for most questions. To simplify reporting in the text, “strongly agree” and “agree” were aggregated into “agree”, and “strongly disagree” and “disagree” into “disagree”.

Licensed pharmacists across Germany were invited to participate in the anonymous online survey through their publicly available email addresses. No reminders were sent or incentives were offered. Reporting follows the “Checklist for Reporting Results of Internet E-Surveys” (CHERRIES).^31^

Although the analysis primarily focused on descriptive statistics, additional statistical tests were conducted using RStudio (version 2022.12.0 + 353). Chi-square and Fisher's exact tests (Monte Carlo approximation, 2000 replicates) were used where appropriate.^20^^,^^32^^,^^33^

### 2.3 Ethics approval

The study's design was approved by the ethics committee of Witten/Herdecke University (reference No. 49/2022) and conducted in compliance with all relevant data protection laws.

## 3 Results

### 3.1 Qualitative interviews

The interviews were conducted between November 11, 2023, and January 15, 2024. After twelve interviews, thematic saturation was observed. Table 1 summarizes interviewee characteristics.

**Table 1:** Demographics of interviewed pharmacists.

ID	Gender	Age group	Pharmacy size		Pharmacy location by city inhabitants	Share of medication distributed outside of pharmacy	Experience with ePs
			By # of locations	By # of employees			
P1	Female	26–35	2 locations	10–12	Fewer than 5000	25–80%	Medium
P2	Male	36–45	2 locations	10–12	5000 to 20,000	5–10%	High
P3	Female	26–35	1 location	10–12	5000 to 20,000	1–5%	High
P4	Female	46–55	3 locations	10–12	20,000 to 100,000	5–10%	Medium
P5	Male	26–35	4 locations	4–6	More than 500,000	5–10%	Low
P6	Female	46–55	2 locations	4–6	20,000 to 100,000	Less than 1%	Low
P7	Female	36–45	2 locations	4–6	100,000 to 500,000	5–10%	Medium
P8	Male	56–65	1 location	10–12	5000 to 20,000	10–25%	High
P9	Female	36–45	1 location	4–6	5000 to 20,000	1–5%	High
P10	Male	>65	2 locations	13–15	20,000 to 100,000	1–5%	Medium
P11	Male	26–35	2 locations	16–19	More than 500,000	1–5%	Low
P12	Female	36–45	1 location	10–12	5000 to 20,000	1–5%	Medium

**Fig. 1:**
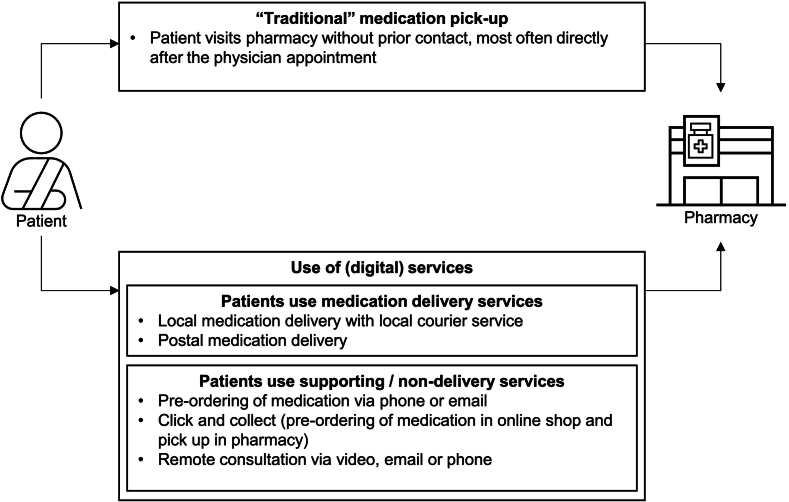
Separation of medication dispensing into “traditional” medication pick-up and the utilization of (digital) services offered by pharmacies: Illustration of how pharmacists interact with patients. Upper part shows “traditional” in-pharmacy pick-up without prior digital interaction, while the lower part depicts (digital) service utilization, including medication delivery and supporting services.

Pharmacists described their core process as medication dispensing, comprising “traditional” medication pick-up in the pharmacy without prior interaction and (digital) service utilization, such as medication delivery services and supporting services (Fig. 1). According to pharmacists, the introduction of ePs had both positive and negative impacts on medication dispensing for pharmacists and patients.

#### 3.1.1 Perceived benefits of ePs

Pharmacists noted that the digital prescription transfer improved medication delivery, particularly postal medication delivery.

“The main benefit of ePs is the digital transfer. Patients can send their prescription directly from their phone, which speeds up postal medication delivery as they don't have to send the prescription via post anymore, but they need special apps to read the health insurance card.”

P10

Furthermore, pharmacists reported fewer prescription errors due to illegible handwriting, missing signatures, or incomplete information. They highlighted automatic validation to help flag inconsistencies in dosage, interactions, or patient data before dispensing.

“Although it didn't happen that often, but with ePs, I no longer need to call the doctor to clarify unreadable handwriting.”

P4

Additionally, administrative and logistical processes, such as billing with health insurance companies or reordering of out-of-stock medications, were also simplified.

“With paper-based prescriptions, a driver typically collected all prescriptions once a week by truck, whereas with ePs, this process is handled digitally.”

P1

These perceived benefits were mainly emphasized by younger pharmacists and those with more experience using digital tools, who viewed ePs as a necessary step toward broader healthcare digitalization.

#### 3.1.2 Perceived challenges of ePs

Despite benefits, 11 of 12 pharmacists described recurring technical problems that disrupted daily workflows, including system crashes and connection issues.

“After redeeming 3-5 ePs, the system usually crashes and I need to restart the Telematik Infrastructure connector (device to establish secure connection to redeem ePs), which takes 5-10 minutes. I can't redeem any ePs during that time.”

P2

Furthermore, pharmacists mentioned delays of up to three hours for ePs to be available in the system, forcing patients to wait or return later.

“The physician around the corner has set their system to bulk signature, where the physician signs 20 ePs at the same time. The system only uploads signed ePs, which sometimes means the eP is not uploaded for 2-3 hours. If patients come directly from the physician and their eP is not uploaded, they need to come back later.”

P9

#### 3.1.3 Community pharmacies specific challenges

Pharmacists mentioned limited digital readiness as a major challenge for themselves. They emphasized that improvements to postal medication delivery primarily benefit large online pharmacies, which have established online presences and processes for postal medication delivery.

“Hosting an online shop is difficult for us. None of us know how to do that, and we are already understaffed. It's difficult to find pharmacists that want to work in this small town. Big [online delivery pharmacies] players have infrastructure and processes in place and can push us out of business.”

P1

Key operational challenges included lack of technical expertise, capacity constraints, and financial barriers. Observed challenges varied for different demographics: Interviewees from rural areas (e.g., P9) emphasized capacity constraints and limited technical infrastructure as main barriers, whereas younger pharmacists and those from urban settings (e.g., P5, P11) focused on financial constraints.

Pharmacists did not observe a significant increase in revenue after introducing medication delivery, citing low patient demand mainly due to perceived long delivery times and limited awareness of digital options.

“Patients usually just stop by the pharmacy on their way home from the physician to pick up the medication.”

P6

“In case of prescription medication, patients usually immediately need the medication (e.g., antibiotics), and as a result rather take a longer drive than wait a day for a postal delivery.”

P8

“I don't think patients are aware of postal medication delivery because, for example, we also don't advertise for it.”

P4

Overall, pharmacists acknowledged that ePs may drive broader pharmacy digitalization, but noted that these developments have not yet fully translated into tangible improvements.

“Once everything runs smoothly, I can imagine ePs will make work easier. But right now, it's just more effort for everyone.”

P5

“I think ePs are the right direction. It just needs time and better support for smaller pharmacies.”

P7

### 3.2 Quantitative online survey

The survey was live from March 4 to April 28, 2024 and yielded 1215 completed responses, with a minor share of “prefer not to answer / I don't know” responses. The sample (Table 2) was largely representative of the German pharmacist population, aligning with national averages in age, location, and pharmacy size.^2^ However, female pharmacists were underrepresented in the sample (57.2% vs. the 2022 national average of 73.6%) and were on average younger than male pharmacists (42.8% of female pharmacists were below the age of 45 years vs. 35% of male pharmacists). Pharmacies with one location were overrepresented (61.4% vs. the national average of 55.4%).^2^

**Table 2:** Demographics of survey.

Characteristic, *n* = 1215	Respondents, n (%)
Gender	
Male	496 (40.8%)
Female	695 (57.2%)
Diverse	24 (2.0%)

Age in years	
< 25	14 (1.2%)
26–35	168 (13.8%)
36–45	297 (24.4%)
46–55	362 (29.8%)
56–65	299 (24.6%)
>65	57 (4.7%)
Prefer not to say / I don't know	18 (1.5%)

Pharmacy size: # of locations	
1 location	746 (61.4%)
2 locations	231 (19.0%)
3 locations	116 (9.5%)
4 locations	93 (7.7%)
Prefer not to say / I don't know	29 (2.4%)

Pharmacy size: # of employees	
1–3	92 (7.6%)
4–6	287 (23.6%)
7–9	339 (27.9%)
10–12	197 (16.2%)
13–15	106 (8.7%)
16–19	72 (5.9%)
20 or more	87 (7.2%)
Prefer not to say / I don't know	35 (2.9%)

Pharmacy location by city inhabitants	
<5.000	169 (13.9%)
5.000–20.000	423 (34.8%)
20.001–100.000	298 (24.5%)
100.001–500.000	168 (13.8%)
>500.000	141 (11.6%)
Prefer not to say / I don't know	16 (1.3%)

Medication delivery license	
Yes	325 (26.7%)
No	847 (69.7%)
Prefer not to say / I don't know	43 (3.5%)

Share of medication distributed outside of pharmacy	
<1%	257 (21.2%)
1–5%	434 (35.7%)
5–10%	244 (20.1%)
10–25%	160 (13.2%)
25%–80%	55 (4.5%)
>80%	4 (0.3%)
Prefer not to say / I don't know	61 (5.0%)

#### 3.2.1 General attitudes toward ePs

Nearly half of pharmacists (49.3%, 599/1215) supported the transition to ePs (agreed with “I approve the transition from paper-based prescriptions to ePs”), while 37.0% (450/1215) disagreed. Support was higher among female pharmacists (53.4%, 371/695) than male pharmacists (43.8%, 217/496), and among younger pharmacists (<36 years, 59.9%, 109/182 in favor) compared to those over 55 years (41.3%, 147/356, ${\chi^{2}}_{20}$= 41, *P* < .005, Cramer V = 0.11, 95% CI = 0.08–0.14). Support was higher among pharmacists in larger pharmacies by number of employees (${\chi^{2}}_{24}$= 39, *P* < .05, Cramer V = 0.11, 95% CI = 0.09–0.14) and number of locations (${\chi^{2}}_{12}$= 27, *P* < .01, Cramer V = 0.10, 95% CI = 0.07–0.13). No effect of pharmacists' attitude was observed for pharmacy location by city size (${\chi^{2}}_{16}$= 22, *P* > .1, Cramer V = 0.08, 95% CI = 0.06–0.11).

#### 3.2.2 “Traditional” medication pick-up in the pharmacy

**Fig. 2:**
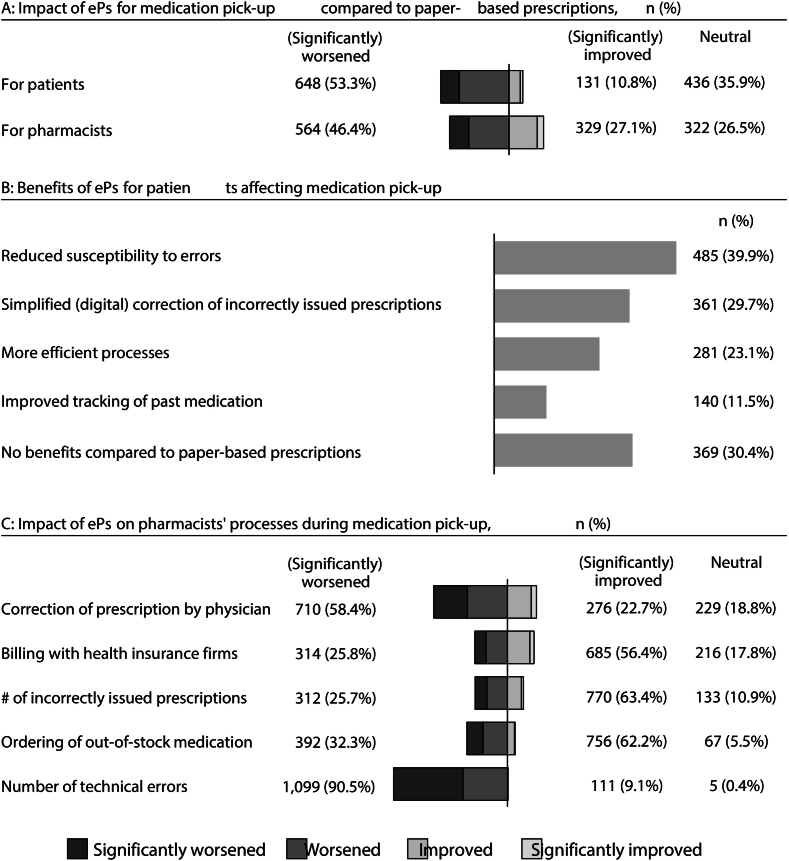
Impact of ePs on traditional medication pick-up and pharmacists' work processes: A: Pharmacists' assessment of impact of ePs on medication pick-up for patients and pharmacists; B: Reported benefits of ePs for patients (multiple-choice question; responses indicate the number of pharmacists selecting each benefit category); C: Impact of ePs on pharmacists' work processes, showing the proportion reporting improvements or worsening in specific areas (e.g., number of technical errors).

More than half of pharmacists (53.3%, 648/1215) believed that ePs worsen medication pick-up in pharmacies for patients, and 46.4% (564/1215) believed ePs worsen pharmacists' workflows (Fig. 2A). Impact on pharmacists' workflows was perceived similarly across genders (47.7% 237/496 of male and 44.8% 312/695 of female). However, male respondents perceived the impact of ePs for patients more negatively (58.4% 290/496 of male and 49.0% 314/695 of female). Pharmacists who were more positive about ePs (based on the statement “I approve the transition from paper-based prescriptions to ePs”) perceived changes during medication pick-up in the pharmacy more positively for both patients and themselves (${\chi^{2}}_{16}$=348–449, *P* < .001, Cramer V = 0.27–0.30, 95% CI = 0.24–0.30 & 0.28–0.34).

The worsening of medication pick-up for patients could stem from low perceived benefits of ePs, as 30.4% (369/1215) of pharmacists saw no benefits (Fig. 2B). Additionally, 81.1% (985/1215) mentioned that digitalization is generally challenging for patients, especially for non-digitally affine patients.

For pharmacists, the worsening of medication pick-up was linked to limited improvements in work processes and increased technical errors (e.g., delayed synchronization or system downtime; Fig. 2C). Pharmacists who experienced fewer technical errors were more positive toward ePs (${\chi^{2}}_{16}$=192, *P* < .005, Cramer V = 0.20, 95% CI = 0.18–0.26). Younger pharmacists perceived more pharmacy workflow improvements than older pharmacists (age in years compared to impact for pharmacists, ${\chi^{2}}_{20}$=37, *P* < .01, Cramer V = 0.10, 95% CI = 0.08–0.13). Additionally, other demographic variables (e.g., city size, number or size of pharmacies) had no significant effect on improvements.

#### 3.2.3 (Digital) service offering

**Fig. 3:**
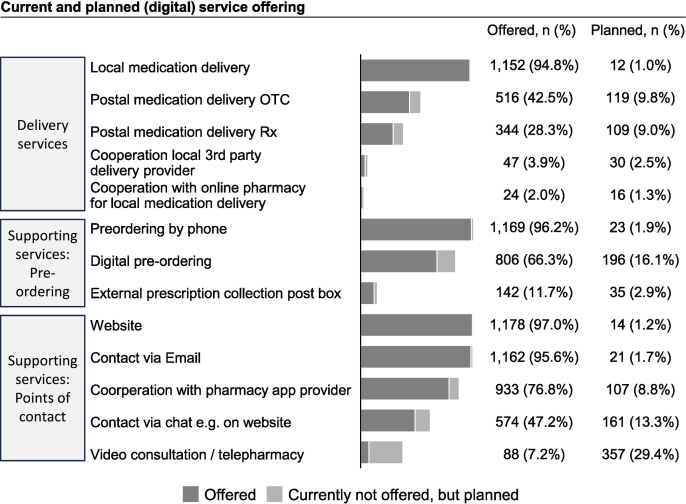
Current and planned (digital) services in German pharmacies: Number of pharmacies that currently offer (dark bars) and plan to offer (light bars) services, grouped into medication delivery services, supporting services for pre-ordering, and supporting services for patient contact. OTC = non-prescription medicines; Rx = prescription medicines.

Fig. 3 shows the current and planned (digital) services of surveyed pharmacies. Most pharmacies (88.1%, 1071/1215) already offered five or more (digital) services, primarily focusing on supporting services (e.g., pre-ordering by phone offered by 96.2%, 1169/1215, website 97.0%, 1178/1215, email contact 95.6%, 1162/1215) and some planned to expand offerings, including digital pre-ordering (16.1%, 196/1215) or video consultation (29.4%, 357/1215).

While most pharmacists reported distributing less than 5% of medication outside of the pharmacy (59.9%, 691/1154), and only 5.1% (59/1154) dispensed more than 25% externally (Table 2), almost all pharmacies (94.8%, 1152/1215) offered local courier medication delivery, with little variation across urban and rural settings. Postal medication delivery was less common, offered by 42.5% (516/1215) for over-the-counter medication (OTC) and 28.3% (344/1215) for prescription medication (Rx). Fewer than 10% planned to introduce postal medication delivery (9.8%, 119/1215 for OTC and 9.0%, 109/1215 for Rx).

#### 3.2.4 Impact of ePs on (digital) services for patients

**Fig. 4:**
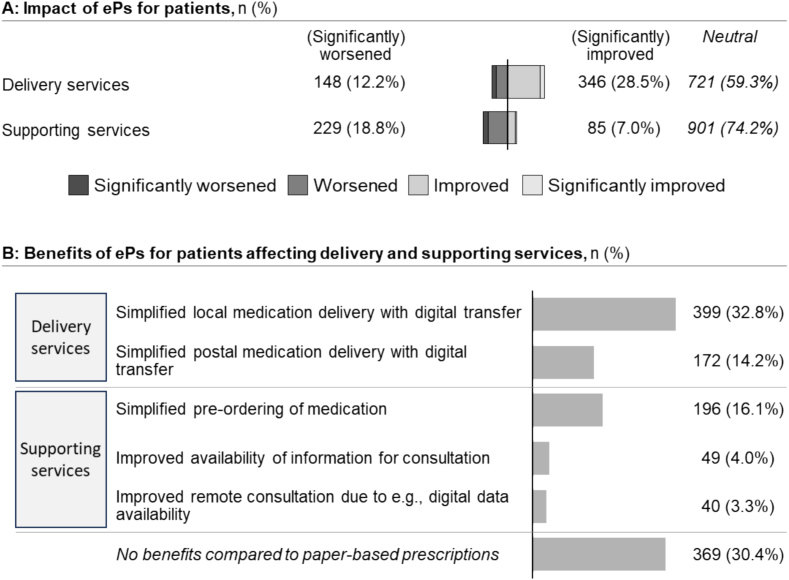
Impact and perceived benefits of ePs for patients on (digital) services: A: Pharmacists' assessments of ePs' potential to improve or worsen delivery services and supporting services for patients; B: Reported benefits of ePs for patients (multiple-choice question; responses indicate the number of pharmacists selecting each benefit category).

Most pharmacists (59.3%, 721/1215) assessed the impact of ePs on medication delivery for patients as neutral and 28.5% (346/1215) reported improvements (Fig. 4A). Age or gender had little effect on perceived improvements (e.g., 29.4%, 146/496 of male vs. 28.4%, 198/695 of female pharmacists reported improvements).

Improvements for patients were mainly driven by the digital transfer of ePs (Fig. 4B). While 32.8% believed this improves local medication delivery, only 14.2% believed it simplifies postal medication delivery. Pharmacists who were more positive toward ePs also perceived greater benefits for patients (${\chi^{2}}_{16}$=223, *P* < .005, Cramer V = 0.21, 95% CI = 0.19–0.25).

#### 3.2.5 Impact of ePs on (digital) services for pharmacists

**Fig. 5:**
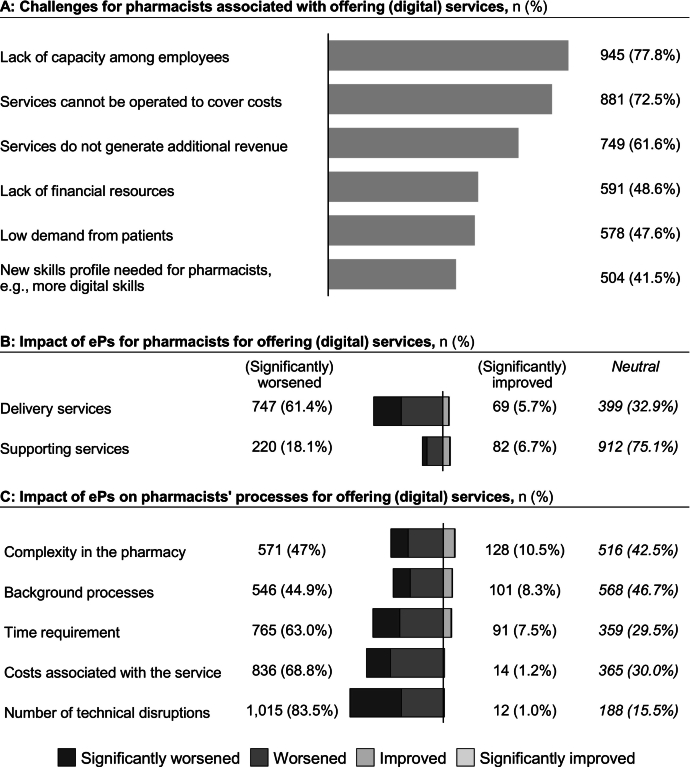
Challenges and impacts of ePs on pharmacists' services and workflows: A: Reported challenges of offering (digital) services (multiple-choice question; responses indicate the number of pharmacists selecting each category); B: Impact of ePs on (digital) services for pharmacists, showing the proportion reporting improvements or worsening; C: Impact of ePs on pharmacists' work processes, showing the proportion reporting improvements or worsening in specific areas (e.g., complexity in the pharmacy).

Offering (digital) services was generally seen as challenging for pharmacies (Fig. 5A): 77.8% (945/1215) mentioned limited staff capacity, 61.6% believed these services do not generate additional revenue (749/1215) and 72.5% believed these are not profitable (881/1215). This was linked to increased cost (68.8%, 836/1215) and low patient demand (47.6%, 578/1215).

The results indicated that ePs do not improve (digital) services for pharmacists, as 61.4% (747/1215) reported that medication delivery services worsened with ePs (Fig. 5B). This was linked to workflow inefficiencies, technical issues (see Section 3.2.2), increased costs, and staff capacity needs (Fig. 5C). While gender had minimal influence on perceptions, younger pharmacists were less negative: 50.5% (92/182) of those under 35 reported worsening compared with 64.4% (241/374) of pharmacists over 55. Pharmacists who were more positive about ePs reported fewer negative effects (${\chi^{2}}_{16}$=358, *P* < .005, Cramer V = 0.27, 95% CI = 0.25–0.34). Despite drawbacks, pharmacists cited increased customer attractiveness (63.4%, 770/1215) and competition from online pharmacies (56.9%, 691/1215) as reasons for offering services.

## 4 Discussion

In this study, qualitative interviews and an online survey were used to explore pharmacists' experiences with ePs and their potential to improve patients' access to medication in Germany, focusing on medication dispensing. By combining both qualitative interview narratives and quantitative survey results, this study provides a more comprehensive understanding of pharmacists' acceptance, use patterns, and perceived benefits and challenges of ePs.

While extant literature has concluded that ePs can improve access to medication both during “traditional” medication pick-up and through (digital) services offered by pharmacies, especially medication delivery,^8^, ^9^, ^10^ the findings of this study indicate that this potential remains largely unrealized in Germany due to technical, economic, and patient-related barriers.

### 4.1 Technical maturity of eP system

Surveyed pharmacists reported frequent technical errors (e.g., system crashes) affecting both medication pick-up and (digital) services, consistent with previous, international research.^34^^,^^35^ Interview data provided concrete examples of how these disruptions affected workflows and patient experience (e.g., repeated restarts or delayed prescription uploads that forced patients to return later). Even minor disruptions increased workload and reduced willingness to engage with (digital) services. Hence, technical maturity appears to reduce performance expectancy and increase effort expectancy, negatively affecting technology adoption, in line with the predictors of acceptance discussed in the Unified Theory of Acceptance and Use of Technology (UTAUT) Framework.^22^

These challenges reflect the early stage of Germany's eP implementation. International comparisons show that countries like Sweden and Estonia, with more mature eP systems, experienced fewer disruptions, allowing more seamless workflows and tangible benefits for patients.^13^^,^^36^ Technical errors tend to decrease over time as systems mature.^15^ Hence, ongoing improvements by the Gematik, the organization responsible for the eP rollout in Germany, could improve prescription redemption for patients and pharmacists in the future.^37^

### 4.2 “Traditional” medication pick-up

While the qualitative interviews suggested that ePs have a limited effect on “traditional” medication pick-up for patients as the general process is unchanged, most surveyed pharmacists reported a worsening for patients. Pharmacists also reported worsened work processes for themselves. According to UTAUT, perceived usefulness depends both on patient outcomes and users' workflow experience. High effort expectancy due to disruptions diminishes perceived usefulness,^22^^,^^23^^,^^38^ which may explain why pharmacists in the survey associated limited benefits with ePs, despite extant literature identifying multiple.^15^^,^^39^^,^^40^

Nonetheless, pharmacists in both interviews and the survey observed a reduction in medication errors consistent with literature,^41^ mainly due to automated validation and the elimination of illegible handwriting. Pharmacists who experienced workflow improvements also reported greater improvements for patients, aligning with existing technology adoption literature.^42^

### 4.3 (Digital) services

Both the interviews and the survey confirmed that pharmacists in Germany perceived offering (digital) services as challenging, which is in line with literature.^43^ Key barriers included financial drawbacks, low patient demand, and limited staff capacity, particularly in smaller pharmacies.

#### 4.3.1 Drawbacks for pharmacists

High perceived drawbacks for users are a driver of overall low perceived usefulness of digital healthcare applications.^22^^,^^44^ Beyond technical errors (Section 4.1), pharmacists associated (digital) services with economic drawbacks.

Operating economically is essential for pharmacies to secure their own survival, and most pharmacists did not expect (digital) services to generate revenue or to increase profitability. Many feared that ePs would strengthen large online delivery pharmacies, perceived as the main competitors to traditional German community pharmacies. These competitors were already able to generate revenue, and some also profit from medication delivery,^45^ potentially due to better economies of scale.^46^ The digital transfer of ePs could further improve their business model.^47^ Nonetheless, ePs have driven increased digitalization of community pharmacies in other countries (e.g., introduction of automatic medication pick-up stations in Finland).^48^

Pharmacists also mentioned missing technical knowledge and digital capabilities. Younger pharmacists showed more positive attitudes toward ePs and (digital) services compared to older pharmacists, consistent with evidence that digital affinity decreases with age among healthcare professionals.^22^^,^^49^ This likely reflects differences in digital literacy rather than structural differences between pharmacies.

#### 4.3.2 Patient demand

Most pharmacists in the survey experienced limited patient demand for (digital) services, an important factor for perceived usefulness of digital healthcare technologies.^22^^,^^42^^,^^50^

The limited demand may be linked to the fact that not all patients were technically able to digitally transfer their ePs, mostly due to the lack of a health insurance card with a Near Field Communication (NFC) tag. In Germany, the health insurance card was historically not equipped with an NFC tag; however, an NFC-enabled card is required as a digital authentication tool that allows patients to securely access, store, and transmit electronic prescriptions.^51^ Other countries did not face this issue because e-prescription infrastructures differ; for example, Sweden introduced patient smart cards used for ePs as early as the 1980s.^13^

Additionally, at the time of the study, many physicians had nearby pharmacies, allowing patients to pick up prescriptions immediately^3^ and reducing the need for medication delivery services, as mentioned also by interviewed pharmacists. However, if pharmacy closures continue,^5^ patient demand for (digital) services may increase. Nonetheless, interest in medication delivery has increased,^52^^,^^53^ which might explain why some pharmacies plan to introduce medication delivery services.

These patient-related barriers are consistent with UTAUT constructs of social influence and facilitating conditions, emphasizing the importance of patient readiness for successful eP adoption.^22^

### 4.4 Levers to improve ePs

Based on these findings, the main lever to realize ePs' potential to improve access to medication is reducing technical errors, as improved technical stability enhances perceived usefulness.^38^^,^^44^ Furthermore, pharmacists play a key role in expanding (digital) services and raising patient awareness.^42^ Policymakers could address pharmacists' challenges by ensuring economic viability of (digital) services and providing targeted digital trainings, particularly for older pharmacists. Ensuring patients' access to ePs' full functionality, including NFC-ready health insurance cards, is essential.^51^

### 4.5 ePs potential beyond the pharmacy

While the points discussed above show that ePs currently do not improve access to medication during dispensing, ePs might nonetheless improve access during other steps of the patient journey. Potential benefits include a reduced need for in-person visits for follow-up prescriptions^12^ and the facilitation of digital healthcare applications like electronic patient records.^54^ Findings from other countries concluded that the introduction of ePs has expanded digital healthcare service offerings for patients.^55^, ^56^, 57.

(Digital) services also influence patient counseling and monitoring. Although medication delivery could reduce face-to-face counseling opportunities, AI-assisted tools and integrated digital systems could support adherence monitoring and personalized care.^58^^,^^59^

### 4.6 International relevance

While this study focuses on the German context, its findings on ePs in pharmacies, as well as the used methods contribute to international technology acceptance research. Similar challenges identified in this study, have also been reported internationally during early phases of eP implementation.^60^^,^^61^ The study therefore provides transferable insights for policymakers and health system planners in other countries, for example by identifying levers to improve eP implementation in pharmacy settings.

In addition, similarities can be observed across the rollout of different digital healthcare technologies.^20^^,^^62^ The identified drivers and barriers are thus relevant not only for countries implementing ePs, but also for those introducing other digital healthcare tools, such as video consultations. Moreover, the methods used in this study offer a transferable framework that can be adapted for cross-national research and contribute to the broader international technology acceptance literature.

Additionally, the observed tension between community pharmacies and large online pharmacies reflects global trends in pharmacy digitalization.^15^ While market concentration is a common outcome of digitalization, it raises concerns in healthcare.^63^ Germany's experience shows that, despite regulatory safeguards,^16^ small community pharmacies may struggle to remain competitive. These findings highlight a potential risk for healthcare systems and may help policymakers in other countries anticipate unintended consequences of digitalization and consider measures to support the long-term viability of community pharmacies.^48^

### 4.7 Limitations and future research

While the methodology was developed with a focus on accuracy and reliability, natural limitations exist. First, at the time of the study, the number of patients using ePs was relatively low, making licensed pharmacists the most appropriate respondents. Consequently, the results might differ if patients were surveyed directly, particularly regarding improvements for patients resulting from the introduction of ePs. With increasing eP adoption, future research should incorporate patient perspectives.

Second, while the large sample size strengthens the study, self-selection bias may exist due to voluntary participation of pharmacists.^12^ Email-based recruitment may have underrepresented less digitally active pharmacies, resulting in a sampling or response bias.^64^ Furthermore, pharmacists from large online pharmacies were underrepresented, and their experiences may differ given the different business model.^14^ Future research should address this gap. Furthermore, while the sample includes pharmacists from different city sizes, the typically close proximity to physicians may have influenced perceptions of delivery services.

Third, the interviews were documented via extensive note-taking, which may have led to a loss in detail compared to audio recording and verbatim transcription.^65^ Consequently, while major topics were captured, some nuances may have been missed. The structured interview guide was used as a tool to mitigate reliability issues.

Furthermore, the use of a five-point Likert scale may have introduced a central tendency bias, where survey participants avoid extreme categories and instead cluster their responses around the midpoint.^66^ Additionally, the aggregation of categories for readability could have reduced variability between “strongly” and “somewhat” responses.

Finally, the findings indicate that while the potential of ePs to enhance medication access has not yet been fully realized, future technical improvements, increased patient demand, and rapid policy or infrastructure changes may make the findings time-sensitive. As a result, future studies could replicate this approach to monitor and analyze these developments.

### 4.8 Conclusion

The findings suggest that while ePs have the potential to improve access to medication, particularly through (digital) services, this potential was limited in Germany due to technical instability, low patient digital literacy, and pharmacists' perceptions of limited usefulness. With continuing pharmacy closures, (digital) services like medication delivery might become increasingly important.

To realize the full potential of ePs, concrete policy measures are needed, including funding programs for small pharmacies, nationwide technical assistance, and targeted training campaigns for older pharmacists. Equally important is improving patient digital readiness, as low digital literacy remains a key barrier.

Looking ahead, the increasing maturity of ePs and growing consumer readiness may allow (digital) services to evolve from basic medication delivery options toward integrated care solutions (e.g., chronic care management and personalized medication support).^67^ More broadly, ePs play a key role in healthcare digitalization beyond medication dispensing.

In conclusion, realizing the full potential of ePs requires technical reliability, economic viability of (digital) services, and improved digital literacy for pharmacists and patients.

## Summary table

What was already known on the topic:

• ePs have been observed to improve access to medication for patients
• ePs have led to an increase in (digital) service offerings, benefiting patients
• Perceived usefulness of a healthcare application is linked to perceived benefits and drawbacks, effort expectancy and patient demand

What this study added to existing knowledge:

• In Germany, the potential of ePs to improve access to medication is currently unrealized due to frequent technical errors and low perceived usefulness by pharmacists
• While the digital transfer of ePs is seen as a benefit for benefit for patients, limited other improvements of ePs in Germany have been observed
• ePs worsen pharmacists' work processes and are perceived as a threat to traditional pharmacies due to increased competition from large online delivery pharmacies
• The main levers to unlock ePs' potential to improve access to medication in Germany are reducing technical errors, ensuring economic viability of (digital) services for pharmacists and enabling all patients to use ePs' full technical functionality

## CRediT authorship contribution statement

**Alexander Graf:** Writing – original draft, Visualization, Validation, Methodology, Investigation, Formal analysis, Conceptualization. **Maike Henningsen:** Writing – review & editing, Methodology, Conceptualization. **Maximillian Zinner:** Writing – review & editing, Methodology, Conceptualization.

## Declaration of competing interest

The authors declare that there is no conflict of interest.

## Figures and Tables

**Fig. A.1:**
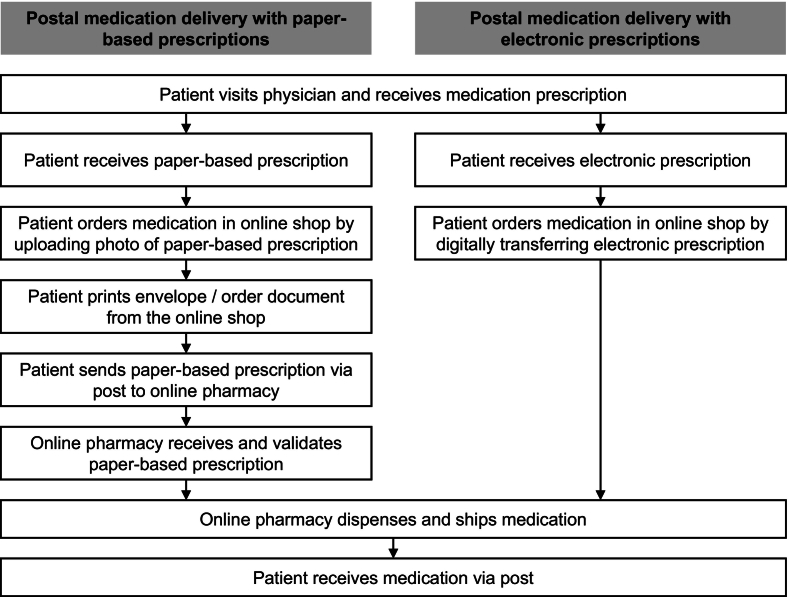
Comparison of traditional and eP-based postal medication delivery: Flow chart contrasts the traditional postal delivery workflow using paper prescriptions (left) with the digital process enabled by ePs (right).

**Fig. A.2:**
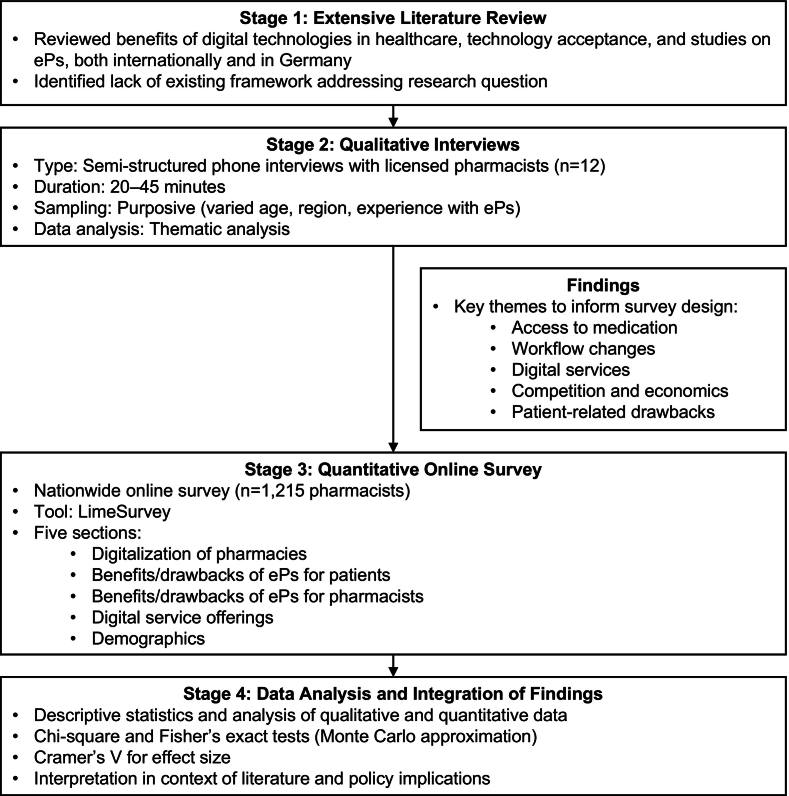
Study design and methodological flow: Overview of the sequential mixed-methods approach used in this study, beginning with a literature review, followed by qualitative interviews with pharmacists and thematic analysis. Findings from the interviews informed the development of an online survey. Quantitative data were analyzed using Chi-square tests and Cramer’s V, and results were analyzed to answer the research question.

**Table:** 

	Offered	Currently not offered, but planned	Currently not offered and not planned	No answer
Local delivery	○	○	○	○
Postal medication delivery OTC	○	○	○	○
Postal medication delivery Rx	○	○	○	○
Cooperation local 3rd party delivery provider	○	○	○	○
Cooperation with online pharmacy for local delivery	○	○	○	○
Preordering by phone	○	○	○	○
Digital pre-ordering	○	○	○	○
External prescription collection post box	○	○	○	○
Website	○	○	○	○
Contact via Email	○	○	○	○
Cooperation with pharmacy app provider	○	○	○	○
Contact via chat (e.g., on website)	○	○	○	○
Video consultation / telepharmacy	○	○	○	○

**Table:** 

	Daily	Weekly	Monthly	Less than monthly	Never	No answer
Electronic prescriptions	○	○	○	○	○	○
Digital pre-ordering of medication	○	○	○	○	○	○
Local medication delivery	○	○	○	○	○	○
Postal medication delivery	○	○	○	○	○	○

**Table:** 

	Highly improved	Improved	No effect	Worsened	Highly worsened	No answer
“Traditional” medication pick-up	○	○	○	○	○	○
Delivery services	○	○	○	○	○	○
Supporting services	○	○	○	○	○	○

**Table:** 

	Highly improved	Improved	No effect	Worsened	Highly worsened	No answer
Overall	○	○	○	○	○	○
Correction of prescription by physician	○	○	○	○	○	○
Billing with health insurance companies	○	○	○	○	○	○
Number of incorrectly issued prescriptions	○	○	○	○	○	○
Ordering out of stock medication	○	○	○	○	○	○
Number of technical errors	○	○	○	○	○	○

**Table:** 

	Highly improved	Improved	No effect	Worsened	Highly worsened	No answer
Overall delivery services	○	○	○	○	○	○
Overall supporting services	○	○	○	○	○	○
Complexity in the pharmacy	○	○	○	○	○	○
Background processes	○	○	○	○	○	○
Time requirement	○	○	○	○	○	○
Costs associated with the service	○	○	○	○	○	○
Number of technical disruptions	○	○	○	○	○	○

## Appendix A

### A.3 Translated interview guide

The Interview guide was created in German. The questions below are a translation.

• Introduction of interviewer and general research area
• Introduction to interview format
• Information about anonymity, data protection, and data processing
• Could you tell me a bit about yourself, including your professional background and current role?
• How would you currently assess patients' access to medicines in Germany and why?
• How does this differ in rural vs. urban areas?
• Does this affect all medicines or only certain ones?
  ○ Does this primarily affect prescription drugs?
  ○ Does this primarily affect drugs for non-chronic diseases?
• What do you expect for the next years?
• How do patients currently get their medication?
• Which services do you offer?
• Which experiences have you made with electronic prescriptions so far?
• How do electronic prescriptions affect the patient journey for medication dispensing?
• What benefits and drawbacks do you see for patients from ePs compared to paper-based prescriptions?
• Which challenges for patients with ePs have you observed?
• How does the introduction of ePs affect Pharmacists?
  ○ Benefits & Drawbacks?
  ○ Challenges?
• Are there new services you offer / plan to offer because of ePs?
• Anything we forgot / latest comments?

### A.4 Translated survey questionnaire

The questionnaire was presented to respondents in German. This copy was translated, only stating questions included in the publication.

#### Impact of electronic prescriptions on pharmacies in Germany

The nationwide introduction of electronic prescriptions (ePs) is transforming the pharmacy landscape in Germany. This change affects not only patients but especially pharmacists. As part of a research project at Witten/Herdecke University, we are examining the introduction of ePs and aim to shed light on pharmacists' perspectives.

The goal of this survey is to understand the changes in medication dispensing resulting from ePs and to identify barriers and challenges in using ePs. Completing the anonymous survey will take approximately 7-9 minutes. Thank you for your participation and your contribution to research!

This survey is anonymous and does not allow any conclusions to be drawn about your identity. The survey data will be processed solely for scientific research purposes. Participation is voluntary.

By participating, you accept the privacy policy.

The survey consists of 12 content-related questions divided into 4 sections, followed by demographic information.

#### Definition of (digital) services & supporting services in pharmacies

This refers to all services offered in pharmacies beyond the “traditional” medication pick up and do not necessarily need to be digital, such as pre-ordering medication by phone. We consider both services that use ePs as a technology, (e.g., checking availability via the ePs app) as well as unrelated services (e.g., an online shop for over-the-counter medications).

#### Definition of digitalization

In the context of this survey, digitalization includes anything broadly related to the use of digital technologies. This includes, for example, a pharmacy website, an online shop, as well as digital applications like the electronic patient record, the electronic medication plan, or KIM (Communication in Medical Care).

#### Digitalization of pharmacies in Germany

How much do you agree with the statement: I approve the transition from paper-based prescriptions to electronic prescriptions

○ Fully agree
○ Agree
○ Neither
○ Disagree
○ Fully disagree
○ No answer

How much do you agree with the statement: I believe digitalization is useful to patients

○ Fully agree
○ Agree
○ Neither
○ Disagree
○ Fully disagree
○ No answer

How much do you agree with the statement: I believe digitalization is useful to pharmacists

○ Fully agree
○ Agree
○ Neither
○ Disagree
○ Fully disagree
○ No answer

Which digital services do you offer or are planned to offer in the future:

How often are you asked by patients about these topics?

#### Benefits and drawbacks of ePs for patients

Which challenge do you currently see for patients during medication dispensing in Germany?

○ Decline in Pharmacy Density
○ Financial Burden from Medication Co-payments
○ Medication Supply Shortages
○ Digitalization of the Healthcare System (e.g., for Non-Digitally Affine Patients=
○ High Need for Counseling on Usage, Dosage, Side Effects, etc.
○ Limited Pharmacy Opening Hours (e.g., Due to Staff Shortages)
○ Polypharmacy in Patients with Limited Mobility
○ Drug Interactions
○ Lack of Emergency Pharmacies
○ No challenges
○ Other: ___

How do ePs affect the following aspects for patients?

Which benefits do ePs have for patients compared to paper-based prescriptions?

○ Reduced susceptibility to errors
○ Simplified (digital) correction of incorrectly issued prescriptions
○ More efficient prosses
○ Improved tracking of past medication
○ Simplified local delivery due to digital transfer
○ Simplified postal medication delivery due to digital transfer
○ Simplified pre-ordering
○ Improved availability of information for consultation
○ Improved remote consultation due to (e.g., digital data availability)
○ No benefits compared to paper-based prescriptions
○ Other: ___

#### Benefits and drawbacks of ePs for pharmacists

How do ePs affect pharmacists work processes for “traditional” medication pick up?

#### Digital service offerings utilizing ePs

What are reasons for pharmacies to offer (digital) services?

○ Increased customer appeal of the pharmacy
○ Competitive pressure from online and mail-order pharmacies
○ Customer demand for services
○ Opportunity to increase sales
○ Regulatory pressure
○ Local competitive pressure
○ Increased employer attractiveness
○ No reason
○ Other: ___

What challenges do pharmacies have to face for introducing and managing (digital) services?

○ Lack of capacity among employees
○ Services cannot be operated to cover costs
○ Services do not generate additional revenue
○ Lack of financial resources
○ Low demand from patients
○ New skills profile needed for pharmacists (e.g., more digital skills=
○ Lack of training on the topic of digitalization
○ Few ready-made (digital) solutions (e.g., online store via "franchise chain")
○ Lack of offers from cooperation partners (e.g., for delivery service providers)
○ No obstacles
○ Other: ___

How do ePs affect pharmacists work processes for (digital) services?

#### Demographic information

When answering questions regarding aspects such as the **size of the pharmacy**, please refer only to the **branch where you work**. Do not include colleagues working in other branches.

How many locations does your pharmacy “chain” operate?

○ 1
○ 2
○ 3
○ 4

Does your pharmacy own a medication delivery licence?

○ Yes
○ No
○ No answer

How many employees are working in your branch excluding owner?

○ 1-3
○ 4-6
○ 7-9
○ 10-12
○ 13-15
○ 16-19
○ 20 or more

Where is your pharmacy branch located? In a city with …

○ <5.000
○ 5.000-20.000
○ 20.001-100.000
○ 100.001-500.000
○ >500.000 inhabitants

What percentage of Rx medication is handed out outside of the pharmacy?

○ <1%
○ 1-5%
○ 5-10%
○ 10-25%
○ 25%-80%
○ >80%

What is your gender?

○ Male
○ Female
○ Divers
○ No answer

How old are you?

○ < 25
○ 26-35
○ 36-45
○ 46-55
○ 56-65
○ >65

Other comments: ____

Thank you for your contribution to research!
